# Chronic Thromboembolic Pulmonary Hypertension as an Inflammation–Angiogenesis Disorder: From Thrombus Persistence to Dual Pulmonary Vasculopathy

**DOI:** 10.31083/RCM49832

**Published:** 2026-06-25

**Authors:** Christian Biancosino, Ali-Ekber Firat, Bassam Redwan

**Affiliations:** ^1^Department of Thoracic Surgery, Helios University Hospital Wuppertal, University of Witten/Herdecke, 42283 Wuppertal, Germany; ^2^Department of Pulmonology, Knappschaft Kliniken Gelsenkirchen Buer, 45894 Gelsenkirchen, Germany; ^3^Medical Faculty of the Ruhr-University of Bochum, 44801 Bochum, Germany; ^4^Department of Thoracic Surgery, Knappschaft Kliniken Gelsenkirchen Buer, 45894 Gelsenkirchen, Germany; ^5^Faculty of Health & Social Sciences, Fresenius University of Applied Sciences, 65510 Idstein, Germany

**Keywords:** chronic thromboembolic pulmonary hypertension, pulmonary embolism, endothelial cells, angiogenesis inhibitors, extracellular traps, vascular remodeling, pulmonary hypertension

## Abstract

Chronic thromboembolic pulmonary hypertension (CTEPH) is a serious but potentially treatable complication of acute pulmonary embolism. CTEPH is characterized by persistent obstruction of the pulmonary arteries and elevated pulmonary pressure. Although organized blood clots have long been considered the primary cause, recent research indicates that CTEPH is more complex. Indeed, CTEPH encompasses ongoing endothelial dysfunction and dysregulated angiogenic recanalization within organized thrombi. Unlike previous reviews that address these pathways in isolation, this review integrates inflammation and angiogenesis into a unified mechanistic framework, incorporating recent single-cell transcriptomic data and epigenetic findings to outline the development and progression of CTEPH. The review also examines both established and emerging pathomechanisms of CTEPH, focusing on how local blood flow and endothelial activation shape the disease. Moreover, this review highlights the concept of dual vasculopathy, encompassing both significant vessel occlusions and small-vessel changes, similar to those observed in pulmonary arterial hypertension. Additionally, the review examines the role of inflammation in CTEPH, including the involvement of neutrophils, neutrophil extracellular traps, high-mobility group box 1 protein, monocytes, macrophages, and adaptive immune responses, as revealed by single-cell analyses. This review further discusses how endothelial dysfunction is linked to inflammation, thrombosis, and remodeling of the pulmonary vasculature. Particular attention is provided to abnormal von Willebrand factor levels, *NF-κB* signaling, and changes in gene regulation. Impaired angiogenesis appears to be a central mechanism underlying impaired thrombus resolution and the persistence of pulmonary hypertension, as shown in both human and animal studies. Collectively, these findings support the view that CTEPH is fundamentally an inflammatory and angiogenic disorder and suggest novel therapeutic targets that may complement surgery and other interventions.

## 1. Introduction

Chronic thromboembolic pulmonary hypertension (CTEPH) is a major long-term complication of acute pulmonary embolism (PE) [1]. CTEPH frequently results in severe clinical outcomes, particularly right heart failure, owing to persistent obstruction of the pulmonary arteries. These obstructions are composed of organized thrombotic material, increase pulmonary vascular resistance, and, if left untreated, impose pressure overload on the right ventricle [2,3,4]. The inability to resolve thrombi after acute pulmonary embolism leads to dysregulated vascular remodeling, impaired fibrinolysis, and abnormal angiogenesis, ultimately producing fibrous plugs that obstruct vascular pathways [5,6,7]. CTEPH develops in approximately 1–5% of patients after acute PE, and this progression is significantly affected by multiple factors, including hereditary predispositions and comorbid conditions that may lead to a prothrombotic state [3,8]. Incomplete or defective thrombus resolution is critical. At the same time, normal fibrinolytic processes are designed to dissolve blood clots. In CTEPH patients, these mechanisms are often impaired, resulting in fibrotic material that obstructs pulmonary blood flow [9,10]. Additionally, the inflammatory response mediated by monocytes and cytokines is fundamental to the pathogenesis of CTEPH, as it orchestrates thrombus organization and vascular remodeling [11,12]. The clinical presentation of CTEPH is often non-specific, with symptoms such as exertional dyspnea, fatigue, and syncope frequently leading to delayed diagnosis [13,14]. This delay is clinically significant, as prolonged obstruction facilitates the development of a secondary, non-operable small-vessel vasculopathy in non-occluded regions, thereby increasing the risk of residual post-operative pulmonary hypertension. Within the surgical intervention window, as pulmonary endarterectomy (PEA) can be curative, diagnostic delays may determine whether a patient remains operable, underscoring the importance of early screening. Prompt recognition and management are essential for improving patient outcomes, as timely surgery confers significant postoperative survival advantages [8,13]. Current research aims to clarify the molecular mechanisms underlying thrombus persistence and identify novel therapeutic targets to mitigate or reverse CTEPH progression [15]. Importantly, the European Society of Cardiology and European Respiratory Society have introduced the concept of chronic thromboembolic pulmonary disease (CTEPD) without pulmonary hypertension, recognizing that patients may exhibit similar symptoms, perfusion defects, and functional impairment despite normal resting pressures. CTEPD is estimated to be present in approximately 20% of individuals suspected of CTEPH and may benefit from balloon pulmonary angioplasty (BPA) or surgical intervention [16]. An updated systematic review and meta-analysis by Luijten et al. (2023) [17] has refined the epidemiological understanding, with the population-level incidence estimated at approximately 0.56% in all-comers and approximately 3% in PE survivors, while studies using non-catheterization-based diagnosis report higher estimates of up to 6.3%. CTEPH exemplifies the complex interplay between biochemical, cellular, and vascular responses following an acute thrombotic event. Elucidating the mechanisms that regulate thrombus persistence and vascular remodeling is essential for developing preventive strategies and clinical therapies to improve patient outcomes in this debilitating disease.

## 2. Pathomechanisms of CTEPH

Understanding CTEPH requires distinguishing between epidemiological risk factors, which identify predisposed patient populations, and the pathomechanisms that drive disease development at the cellular and molecular level. Risk factors are derived from clinical and epidemiological observations, whereas pathomechanisms—such as inflammatory thrombosis, impaired fibrinolysis, endothelial dysfunction, and defective angiogenesis—are supported by mechanistic *in vivo* and *in vitro* investigations and are discussed in subsequent subsections. The following paragraphs summarize the principal risk factors identified through epidemiological studies. Several hematological risk factors have been associated with CTEPH, including thrombocytosis and elevated factor VIII levels, which contribute to hypercoagulability and thrombus formation [1,18,19]. Autoimmune conditions such as antiphospholipid antibodies or lupus anticoagulant also increase the risk of thrombotic complications post-PE [1,18]. Demographic and historical factors, including advanced age, history of multiple or recurrent pulmonary embolisms, and findings of right heart dysfunction at the time of acute PE diagnosis, also play a significant role [18,20]. Specific patient demographics appear to correlate with an increased likelihood of CTEPH. Studies suggest that while the gender distribution appears balanced in Western registries, significant female predominance (up to a 3.1:1 ratio) has been observed in Japan and other Asian populations, often linked to distinct *HLA* profiles (e.g., HLA-B*5201) and the absence of a history of deep vein thrombosis [21]. Patients tend to be older, averaging about 63 years, and this age factor is consistent across numerous reports [22,23]. The severity of perfusion defects following acute pulmonary embolism has also been identified as predictive of subsequent CTEPH [18]. Additionally, conditions such as inflammatory bowel disease have been recognized as potential drivers of CTEPH development [23,24].

Recent genetic studies have substantially expanded the understanding of CTEPH susceptibility. A genome-wide association study has confirmed shared heritable genetic architecture between deep vein thrombosis, pulmonary embolism, and CTEPH, notably at the *ABO* (*ABO blood group system*) and *FGG* (*fibrinogen gamma chain gene*) loci, with non-O blood groups conferring higher CTEPH risk [25]. Furthermore, Dodson et al. (2025) [26] identified *STAB2* (*stabilin-2 gene*) as a CTEPH risk gene through whole-exome sequencing of high-risk pedigrees. Rare variants in STAB2, encoding *stabilin-2*, a scavenger receptor responsible for clearing the *von Willebrand factor-FVIII *complex, were significantly overrepresented in CTEPH patients compared with PE controls (pooled allele frequency 4.6% vs. 2.2%), with carriers demonstrating elevated plasma *vWF* and factor *VIII* levels [26]. These findings suggest that *STAB2* variants may promote fibrinolysis-resistant thrombi, distinguishing CTEPH genetic susceptibility from classical thrombophilias.

Despite the relatively low incidence of CTEPH following acute PE, it poses considerable morbidity, and approximately 30% of CTEPH patients report no prior history of VTE (venous thromboembolism), underscoring the prevalence of asymptomatic initial events or the possibility of primary pulmonary-specific inflammatory-thrombotic processes [20]. Monitoring for persistent symptoms following acute PE, combined with active assessment of risk factors, may improve the identification of at-risk patients [18,27]. Accordingly, clinical and echocardiographic evaluations are fundamental to implementing post-PE patient care guidelines and recognizing CTEPH at earlier stages [27].

### 2.1 Local Hemodynamics, Endothelial Dysfunction, and Dual Vasculopathy in CTEPH

Local hemodynamics are fundamental to the development and progression of CTEPH. Increased pulmonary vascular resistance is exacerbated by altered shear stress conditions in the pulmonary arteries, which trigger endothelial activation and production of mediators associated with dysregulation [28]. The endothelium, when exposed to varying hemodynamic shear forces, undergoes maladaptive changes manifested by releasing pro-inflammatory and pro-thrombotic factors [29,30]. Shear stress serves as a mechanical stimulus for endothelial cells, regulating their functional responses and influencing processes such as vasodilation via *NO* release. This endothelial dysfunction encompasses a reduction in nitric oxide and prostacyclin bioavailability, alongside a concomitant upregulation of *endothelin-1* and *thromboxane A2*, which collectively promote a pro-constrictive and pro-mitogenic state [29,30]. Importantly, shear stress acts as a double-edged sword in CTEPH. In non-obstructed vascular segments, pathologically elevated shear stress has been shown to induce endothelial-to-mesenchymal transition (EndoMT) through downregulation of the transcription factor *ERG* and reduction of *H3K27ac* enhancer-promoter marks, contributing to occlusive vascular remodeling [31]. Conversely, low shear stress or oscillatory flow downstream of organized thrombi promotes a pro-coagulant endothelial phenotype with increased release of tissue factor and von Willebrand factor. This spatial hemodynamic variance between obstructed and non-obstructed territories underlies the heterogeneous distribution of vascular lesions in CTEPH.

**Fig. 1.**
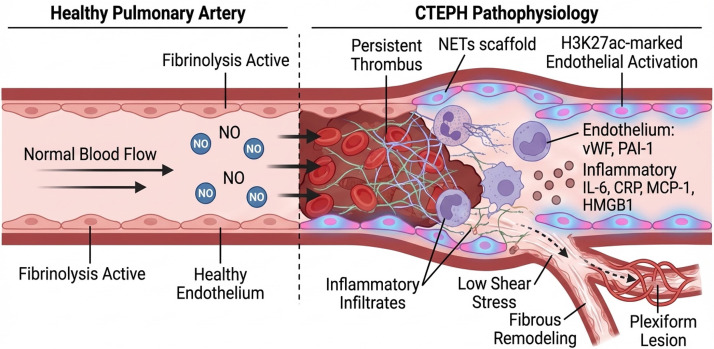
**From acute thromboembolism to dual vasculopathy in CTEPH**. CTEPH, chronic thromboembolic pulmonary hypertension; NETs, neutrophil extracellular traps; NO, nitric oxide; CRP, C-reactive protein; MCP, monocyte chemoattractant protein; HMGB1, high mobility group box; IL, interleukin; PAI-1, plasminogen activator inhibitor-1; vWF, von Willebrand factor; H3K27ac, acetylation of histone H3 at lysine 27.

A crucial aspect of CTEPH is endothelial dysfunction, which not only contributes to disease severity but also serves as a predictive marker for clinical outcomes. Impairment of endothelial function leads to a loss of vasodilatory capacity and an increase in vasoconstrictive responses, which contributes to elevated pulmonary artery pressure [32]. Endothelial dysfunction in CTEPH is further reflected by elevated circulating levels of endothelin-1, a potent vasoconstrictor and smooth muscle cell mitogen, which correlate with hemodynamic severity and may predict adverse outcomes following PEA [33,34,35]. This dysfunction is associated with metabolic changes within endothelial cells, including increased oxidative stress, inflammation, and cell proliferation, all of which promote vascular remodeling [36,37,38]. The presence of a dysfunctional endothelium can activate a series of pathological events, including promoting smooth muscle cell proliferation and further augmenting vascular lesions characteristic of CTEPH (Fig. 1) [32].

From a multimodal management perspective, CTEPH is increasingly conceptualized through a tri-compartmental model, incorporating proximal obstructions, distal microvasculopathy, and the expansion of systemic-to-pulmonary collaterals. The dual vasculopathy comprises both chronic obstruction of the proximal pulmonary arteries by organized thrombi and small-vessel arteriopathy characterized by fibromuscular proliferation, similar to that seen in idiopathic pulmonary arterial hypertension (IPAH) [39,40]. Histopathological analyses have identified small vessel lesions coexisting with organized thrombi, each of which contributes to the progressive nature of the disease [40]. The question of whether plexiform lesions occur in CTEPH remains controversial. Moser and Bloor (1993) [41] reported plexiform-like lesions in a cohort of CTEPH patients based on biopsy and autopsy material; however, it should be noted that PE history was not documented in all cases, and imaging at that time was less sensitive than current modalities. Similarly, Ackermann et al. (2017) [42] described plexiform vasculopathy in a single CTEPH case report. Other investigators, notably Lang et al. (2016) [43], have emphasized that plexiform lesions are typically not found in CTEPH and that histological discrimination between cell-rich colander lesions and true plexiform lesions can be challenging [4]. Accordingly, while the development of PAH-like small-vessel arteriopathy in CTEPH is well established, the presence of plexiform lesions specifically should be regarded as debatable and not yet confirmed by larger systematic studies. Fibromuscular changes in the distal pulmonary arterioles reflect a vascular remodeling process that could be an adaptive

response to altered local hemodynamics and persistent hypoxia, maintaining increased vascular resistance despite the presence of embolic material [39,40]. The drivers of proximal thrombus fibrosis and distal microvascular lesions are increasingly recognized as spatially distinct. Proximal organized thrombi are primarily shaped by impaired fibrinolysis, *NET*-mediated scaffolding, and inflammatory cell infiltration, whereas distal small-vessel arteriopathy is largely driven by redistributed high shear stress, chronic hypoxia, and hemodynamic impact from systemic-to-pulmonary collateral anastomoses. This spatial heterogeneity has important therapeutic implications, as interventions targeting pulmonary endarterectomy (PEA) may not fully address the distal microvascular component.

### 2.2 Microvascular Disease: Where Inflammation and Angiogenesis Converge, CTEPH as a Model Disease

CTEPH serves as a critical model for understanding the interplay between inflammation and angiogenesis in microvascular disease. Evidence from animal and translational models has elucidated how inflammatory processes and angiogenic dysregulation converge, underscoring their roles in the pathogenesis of CTEPH.

### 2.3 Systemic Inflammatory Environment and Circulating Markers in CTEPH

Patients with CTEPH frequently present with elevated levels of pro-inflammatory cytokines such as *interleukin-6* (*IL-6*) and *C-reactive protein* (*CRP*), which have been implicated in disease intensity and advancement [12,44,45]. Notably, elevated *CRP* levels have been correlated with impaired surgical outcomes following pulmonary endarterectomy (PEA), suggesting that systemic inflammation aggravates disease [46]. Inflammation not only modulates the risk of CTEPH, but is also associated with clotting episodes, as elevated pro-inflammatory cytokines indicate a systemic inflammatory state that can affect both thrombus persistence and vasculature [45,47]. Studies indicate that chronic inflammatory conditions such as inflammatory bowel disease and malignancies are associated with an increased risk of CTEPH [2,48,49]. This is also supported by the accumulation of inflammatory cells, such as neutrophils and macrophages, in PEA specimens from patients with CTEPH, demonstrating that persistent inflammation contributes to vascular obstruction and remodeling [48,50]. Inflammation is thought to promote the development of factors that drive pathological angiogenesis and cellular proliferation in the pulmonary vasculature, paralleling changes observed in pulmonary arterial hypertension (PAH) [51,52]. Moreover, there is strong evidence that circulating biomarkers of inflammation and vascular dysfunction, including *matrix metalloproteinases* (*MMPs*) and *IL-10*, are markedly elevated in CTEPH [44,47]. The presence of these markers demonstrates ongoing tissue remodeling and inflammation in the pulmonary vasculature and may serve as potential indicators of disease progression and outcomes [37]. Notably, dysbiosis of the gut microbiota has been suggested to contribute to systemic inflammation by releasing endotoxins, which can activate pro-inflammatory signaling pathways in endothelial cells [53].

## 3. Neutrophils, NETs, and HMGB1 in CTEPH

The pathophysiology of CTEPH is complex and involves neutrophils and the formation of *neutrophil extracellular traps* (*NETs*). The *high mobility group box 1* (*HMGB1*) protein is also implicated in the inflammatory milieu that exacerbates CTEPH, contributing to vascular remodeling and thrombus stability [54].

Neutrophils play a central role in the inflammatory response that is associated with CTEPH. In affected patients, activated neutrophils extrude *NETs*, web-like structures composed of *DNA*, histones, and granule proteins. While these structures trap pathogens, they also contribute to fibrin generation and thrombus formation [55,56]. Circulating neutrophils in patients with CTEPH are often hyperactivated, supporting their involvement in disease pathology [56]. *NETs* facilitate thrombus development and promote fibrous vascular occlusions, underscoring the deleterious impact of neutrophil activity on pulmonary vascular integrity [55,56].

The interaction between *NETs* and *HMGB1* is particularly significant in CTEPH. *HMGB1* acts as a signaling molecule in the immune response and colocalizes with NET-forming neutrophils in thrombi, underscoring its role in the pathophysiology of CTEPH [57,58]. Elevated *HMGB1* levels are observed in related inflammatory diseases, and their presence promotes neutrophil activation and subsequent *NET* release [57,59]. *HMGB1* is considered a potential marker of inflammation and thrombosis, potentially mediating the link between inflammatory processes and thromboembolic events [58,59]. Mechanistically, *HMGB1* functions as a damage-associated molecular pattern (DAMP) that induces process of neutrophil extracellular trap formation (NETosis) through engagement of t*oll-like receptor 4* (*TLR4*) and the *receptor for advanced glycation end products* (*RAGE*) on neutrophils. Both all-thiol and disulfide forms of HMGB1 are capable of triggering NETosis through these distinct receptor pathways, creating a vicious cycle in which *NET*-derived *HMGB1* further amplifies neutrophil activation and platelet aggregation.

The systemic inflammatory environment in CTEPH exacerbates *NET* activation and release. Elevated circulating levels of pro-inflammatory cytokines such as *IL-8* enhance neutrophil recruitment and *NET* formation in patients with CTEPH [44,60]. These mediators perpetuate a cycle of endothelial damage and vascular remodeling, contributing to the chronic and severe nature of pulmonary hypertension [50,61]. The interaction between activated endothelium and neutrophils, which amplifies NETosis, is considered a key factor in the escalation of cellular inflammation and thrombosis [62].

## 4. Monocytes/Macrophages and Adaptive Immunity in CTEPH

Monocytes, precursors of macrophages, are key players in the transition from acute pulmonary embolism to CTEPH. Following embolic events, monocytes are recruited to sites of tissue damage where they differentiate into macrophages that can adopt inflammatory phenotypes [63]. The presence of inflammatory macrophages contributes to the vascular remodeling observed in CTEPH. Studies have suggested that during CTEPH development, monocytes may undergo metabolic reprogramming, regulating their behavior and promoting the formation of atherosclerotic lesions that exacerbate vascular obstruction [63]. Recent experimental approaches, such as metabolic flux analysis, have been used to measure changes in metabolic pathways in monocytes, highlighting reprogramming towards aerobic glycolysis and the pentose phosphate pathway to support cellular biosynthesis and antioxidant defense. These metabolic changes are further supported by upregulation of specific markers such as *glucose transporter 1* (*GLUT1*) and *carnitine palmitoyltransferase 1* (*CPT1*) [47]. The expression of chemokines, such as monocyte chemoattractant protein-1 (MCP-1), has been implicated in the recruitment and activation of these immune cells, thereby accentuating their role in chronic inflammation and pulmonary vascular resistance [47,64]. Additionally, macrophages in CTEPH have been shown to secrete pro-inflammatory cytokines, contributing to a sustained immune response that aggravates pulmonary vascular remodeling. Cytokines such as *interleukin-1β* (*IL-1β*) produced by activated macrophages can increase the inflammatory response, promote cell proliferation, and alter vascular tone thereby exacerbating the hypertensive state [65]. The engagement of these macrophages with other immune cell types, including T cells, creates a milieu conducive to chronic inflammation and may further exacerbate vascular damage [54].

Adaptive immunity also plays a vital role in CTEPH, with evidence indicating that T helper 17 (Th17) cells contribute to the pathogenesis of pulmonary hypertension [54]. The polarization of CD4+ T cells towards an *IL-17*-producing phenotype is relevant, as *IL-17* increases the expression of pro-inflammatory mediators and attracts more immune cells to the inflammatory site, exacerbating the condition [54,66]. Recent studies utilizing single-cell *RNA* sequencing have revealed diverse T cell populations within the thrombus of patients with CTEPH, suggesting the participation of both *CD4+* and *CD8+* T cells in the disease process, consistent with ongoing inflammation and remodeling [54].

Single-cell *RNA* sequencing (*scRNA-seq*) studies have revealed distinctive populations of immune and vascular cells within CTEPH tissues. One study conducted by Chen et al. [63] identified a significant presence of CD16+ monocytes, which localized around areas of thrombus in pulmonary endarterectomy specimens from patients with CTEPH. These observations revealed that monocytes, which can differentiate into macrophages, play a significant role in both inflammatory and fibrotic responses in the pulmonary vasculature, thereby contributing to the pathogenesis of CTEPH.

Miao et al. [50] also contributed to this understanding by demonstrating distinct populations of macrophages and smooth muscle cells (SMCs) within CTEPH lesions and their interactions with endothelial cells.

The presence of multipotent SMC-derived cells capable of differentiating into inflammatory and fibrotic cell types underscores their plasticity and potential to exacerbate pulmonary vascular remodeling. Wang et al. [67] demonstrated that elevated *WNT5B* (*Wnt family member 5B*) expression drives vascular smooth muscle cell dedifferentiation from a contractile to a synthetic/proliferative phenotype through noncanonical Wnt signaling-mediated mitochondrial fission in CTEPH. This metabolic-phenotypic coupling was abolished by mitochondrial division inhibitor 1, while the soluble *Wnt* scavenger *SFRP2* (*secreted frizzled-related protein 2*) attenuated proliferation by promoting mitochondrial fusion, identifying the *WNT5B*-mitochondrial dynamics axis as a potential therapeutic target. Demonstrates the dynamic cellular landscape of CTEPH, where cellular heterogeneity is driven by both pathological and reparative processes.

Notably, a recent landmark single-cell *RNA* sequencing study by Zhang et al. (2025) [68] revealed a dramatic expansion of plasma cells from less than 1% in healthy controls to approximately 15% in CTEPH pulmonary endarterectomy tissue. Five distinct mature plasma cell clusters were identified, including two newly discovered subclusters designated *AHNAK* and *IGLC3*. These plasma cell populations exhibited excessive immunoglobulin production but lacked traditional immune response function, suggesting a dysfunctional humoral immune component in CTEPH pathobiology. Furthermore, immunoglobulin gamma levels were markedly elevated in CTEPH patients compared with both idiopathic pulmonary arterial hypertension patients and healthy controls, pointing toward plasma cells as potential diagnostic biomarkers and therapeutic targets. Additionally, *CRISPLD2*-positive cells, identified through *scRNA-seq* analyses, represent another distinctive cell population in CTEPH tissue, potentially involved in anti-inflammatory signaling within the fibrotic microenvironment [69]. Extracellular vesicles (EVs) have emerged as key mediators of intercellular communication in CTEPH pathogenesis. Leukocyte-derived EVs (LEVs) and endothelial-derived EVs (EEVs) sustain vascular alterations by transferring pro-inflammatory and pro-coagulant cargo between cells, promoting endothelial dysfunction, platelet activation, and microthrombosis [7,70]. Recent translational evidence demonstrated that LEVs and EEVs play a crucial role in sustaining the pulmonary vascular remodeling characteristic of CTEPH, representing a previously underappreciated mechanism in disease progression and a promising target for therapeutic intervention [7].

The interplay between monocytes, macrophages, and adaptive immune cells is integral to forming a feedback loop that perpetuates inflammation within the pulmonary vasculature in CTEPH. This chronic inflammatory state affects not only local vascular cells but may also have systemic consequences, contributing to disease progression and severity [47]. Importantly, these immune mechanisms offer potential targets for mitigating disease progression in CTEPH, underscoring the need to understand the immunological landscape of pulmonary vascular remodeling.

### 4.1 Endothelial Activation and vWF

Endothelial activation is a pivotal process in the development of CTEPH, as it propagates inflammatory processes within the pulmonary vasculature. Activated endothelial cells release various mediators, including *von Willebrand factor* (*vWF*), which plays an essential role in hemostasis and thrombosis. Elevated levels of *vWF* have been correlated with increased pulmonary artery pressures and poor outcomes in patients with CTEPH [71,72]. In CTEPH, a dysfunctional endothelium contributes to the persistent elevation of vWF, amplifying thrombus formation and impairing proper thrombus resolution [72].

### 4.2 NF-κB Signaling

The *nuclear factor kappa-light-chain-enhancer of activated B cells* (*NF-κB*) signaling pathway is a central regulator of inflammation and endothelial function. In CTEPH, *NF-κB* activation contributes to the chronic inflammatory state observed in the pulmonary vasculature. Specifically, *NF-κB2* has been implicated in facilitating *vWF* transcription by promoting *H3K27ac *at the promoter region, a modification that occurs concurrently with a reduction in H3K27me3, establishing a local pro-thrombotic endothelial environment [73]. This epigenetic modification enhances *NF-κB* binding to the *vWF* promoter, thereby facilitating transcription of this pro-thrombotic factor. This regulatory action underscores how persistent *NF-κB* activation drives pathological changes in endothelial cells, leading to ongoing inflammation and vascular occlusion [46]. Furthermore, inflammatory stimuli such as *CRP* have been shown to significantly upregulate *NF-κB* signaling, drive endothelial activation, and increase *vWF* secretion in endothelial cells derived from CTEPH patients [46,51]. This elevation in *vWF* and other adhesion molecules promotes further leukocyte recruitment. This in turn enhances the thrombotic milieu within the pulmonary vasculature, thereby propagating the cycle of endothelial dysfunction and vascular remodeling.

### 4.3 Epigenetic Reprogramming

Epigenetic mechanisms substantially modulate the expression of genes related to vascular health in CTEPH. Studies have demonstrated that aberrant histone modifications, including reduced tri-methylation at *H3K27* (*H3K27me3*) and increased acetylation (*H3K27Ac*) of the *vWF* promoter, are characteristic of CTEPH-derived endothelial cells [73]. Such epigenetic alterations facilitate the transcriptional activity of proinflammatory genes, including *vWF.* The association between *NF-κB2* and epigenetic modifiers is an essential nexus of signaling events that promote a pro-inflammatory and pro-thrombotic endothelial phenotype in CTEPH [73]. Additionally, macrophages and T cells that infiltrate the pulmonary vasculature in CTEPH release cytokines that may alter endothelial cell epigenetic changes, thereby exacerbating inflammatory responses and contributing to vascular remodeling [50]. Understanding the epigenetic landscape of endothelial cells in CTEPH may yield insights into novel therapeutic strategies to reverse pathological changes and restore normal endothelial function.

### 4.4 Impaired Angiogenesis in CTEPH

**Fig. 2.**
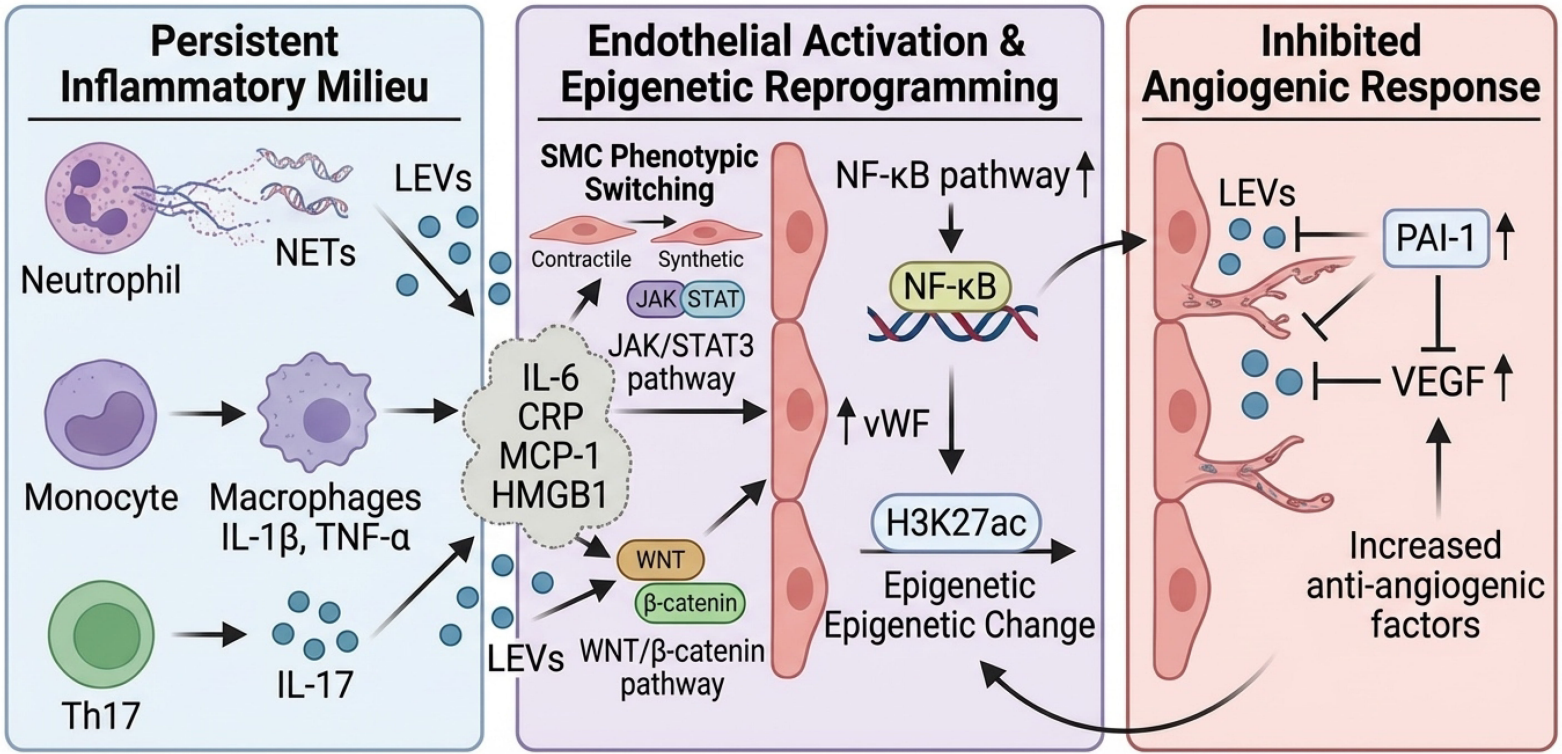
**Inflammatory-angiogenic crosstalk driving CTEPH progression**. NETs, neutrophil extracellular traps; LEVs, leukocyte-derived extracellular vesicle; IL-1β, interleukin-1 beta; Th17, T helper 17 cell; IL-17, interleukin-17; NF-κB, nuclear factor kappa-light-chain-enhancer of activated B cells; WNT, wnt family member; H3K27ac, acetylation of histone H3 at lysine 27; vWF, von Willebrand factor; VEGF, vascular endothelial growth factor; PAI-1, plasminogen activator inhibitor-1.

While CTEPH-derived endothelial cells demonstrate high intrinsic angiogenic potential *in vitro* (mediated by *HGF*), their functional recanalization capacity *in vivo* is constrained by the anti-angiogenic and fibrotic microenvironment, characterized by high levels of *PAI-1* and *angiostatin*. For instance, Tura-Ceide et al. [74] highlighted a hyperproliferative yet dysfunctional endothelial cell phenotype in CTEPH, characterized by diminished angiogenic capabilities and increased expression of adhesion molecules. These dysfunctional endothelial cells are unable to migrate effectively or form new vascular structures, which are crucial for resolving the thrombus and restoring normal pulmonary perfusion [75]. Additionally, investigations of endothelial progenitor cells (EPCs) in CTEPH have revealed impaired neovascularization properties. Yamamoto et al. [76] reported that EPCs stimulated by riociguat might improve angiogenesis, suggesting that EPC dysfunction contributes to the poor vascular recovery observed in these patients. The overall inadequate response in forming new blood vessels is further compounded by elevated levels of *plasminogen activator inhibitor-1* (*PAI-1*), which inhibits angiogenesis by disrupting the *vascular endothelial growth factor* (*VEGF*) signaling pathway (Fig. 2) [77,78].

### 4.5 Mechanistic Insights Into Defective Angiogenesis

The pathways underlying defective angiogenesis in CTEPH can be traced to both local endothelial dysfunction and systemic inflammatory responses. Quarck et al. [79] elucidated that the impairment of angiogenesis-driven clot resolution is a major contributor to the progression to CTEPH, drawing parallels with dysfunctions observed in models of venous thrombosis. The authors highlighted how defective angiogenic responses render the pulmonary vasculature susceptible to persistent obstruction, creating a vicious cycle of vascular remodeling and elevated pressure.

Moreover, the pathophysiological environment created by chronic inflammation within the CTEPH contributes to defective angiogenesis. Markers of inflammation, such as *CRP* and various cytokines, exacerbate endothelial dysfunction, thereby impairing the delicate equilibrium required for effective neovascularization [47]. Angiogenic factors, while capable of promoting initial reparative processes, may fail to elicit organized vessel growth in the persistently inflamed and fibrotic milieu characteristic of CTEPH.

## 5. Impact of Inflammation and Microenvironment

The inflammatory microenvironment in PEA specimens also contributes to the dysregulation of angiogenesis. Chronic inflammation results in the secretion of pro-inflammatory cytokines and chemokines such as *interleukin-6* (*IL-6*) and monocyte *chemoattractant protein-1* (*MCP-1*), which can further exacerbate the presence of anti-angiogenic factors [44,45]. The balance between pro- and anti-angiogenic signals is disrupted in CTEPH, leading to the dominance of inhibitory signals and failure of vascular formation.

### 5.1 Endothelial Dysfunction

Endothelial cells in the PEA material exhibit dysfunctional properties characterized by decreased responsiveness to angiogenic stimuli and increased production of anti-angiogenic factors [77]. This dysfunction significantly affects the vascular repair and thrombus resolution. Importantly, there is no current consensus regarding the angiogenic properties of endothelial cells derived from PEA specimens. While Tura-Ceide et al. [74] reported impaired angiogenic capacity with a hyperproliferative yet dysfunctional phenotype, and Viswanathan et al. [54] observed reduced angiogenic capacities, Naito et al. [52] demonstrated that although endothelial cells derived from PEA material show considerable angiogenic potential, the surrounding pathological milieu may impair angiogenic function. This heterogeneity likely reflects differences in the origin and microenvironment of isolated cells, suggesting that the angiogenic phenotype of CTEPH endothelial cells is context-dependent. Consequently, this contributes to the persistence of pulmonary hypertension despite the surgical removal of occlusive thrombi.

### 5.2 Defective Angiogenesis in Experimental Models

Studies using animal models have demonstrated that angiogenesis is impaired in the context of CTEPH. Quarck et al. [79] developed a rabbit model that mimicked human CTEPH and found that impaired angiogenesis was a key event in disease progression. Their findings demonstrated that inhibition of intrathrombus angiogenesis via *SU5416*, a *VEGF* receptor tyrosine kinase inhibitor, led to elevated pulmonary artery pressure, increased pulmonary vascular resistance, and right ventricular hypertrophy, validating the critical role of local angiogenesis-driven clot resolution in CTEPH progression. Notably, the combination of intrathrombus angiogenesis inhibition via *SU5416* led to sustained elevations in pulmonary pressure, underscoring the significant role of angiogenic processes in maintaining normal pulmonary hemodynamics. Zagorski et al. [80] verified these observations in a rat model of chronic thromboembolic pulmonary hypertension, highlighting that modulation of the soluble guanylate cyclase pathway could ameliorate pulmonary hypertension by stimulating angiogenesis, revealing possible therapeutic means for managing CTEPH by promoting vascular growth. Evidence from animal models thus suggests that angiogenesis-driven clot resolution is a local, intrathrombus process essential for preventing CTEPH progression. While these findings highlight the importance of preserving local angiogenic capacity, therapeutic strategies aimed at promoting vascular growth must be approached with caution, as systemic pro-angiogenic interventions could have unintended consequences. The development of targeted approaches to enhance intrathrombus angiogenesis warrants further investigation rather than simply restoring normal vascular physiology.

### 5.3 Role of Growth Factors and Inhibitors

Translational studies using human pulmonary endarterectomy (PEA) specimens have shown that factors such as *hepatocyte growth factor* (*HGF*) are elevated in patients with chronic thromboembolic pulmonary hypertension. Naito et al. [52] demonstrated that endothelial cells (ECs) derived from PEA specimens exhibited high angiogenic potential, with elevated *HGF *expression, suggesting the possibility of using *HGF* or *HGF* mimetics as pro-angiogenic therapies. This is consistent with the hypothesis that factors that support angiogenesis can facilitate thrombus resolution, thereby improving patient outcomes. In contrast, the presence of anti-angiogenic factors, such as angiostatin and endostatin, has been implicated in CTEPH pathology. Bochenek et al. and Alias et al. [15,77] reported elevated expression of these anti-angiogenic factors in endothelial cells isolated from PEA materials, reinforcing the hypothesis that an imbalance between pro-angiogenic and anti-angiogenic signals contributes to CTEPH. Thus, the regulation of angiogenic signals is complex, and therapies designed to correct existing imbalances may improve angiogenesis and, consequently, vascular health in patients with CTEPH. It is important to note that the angiogenic imbalance in CTEPH is compartment-specific. While intrathrombus and pulmonary vascular angiogenesis is impaired, the bronchial arterial circulation typically exhibits hyperactive compensatory angiogenesis. Systemic-to-pulmonary collateral anastomoses via dilated bronchial arteries develop in response to pulmonary artery occlusion, maintaining distal lung perfusion but contributing to hemoptysis and additional hemodynamic complexity. This compartmental divergence between impaired pulmonary angiogenesis (characterized by high *PAI-1*) and compensatory systemic neovascularization must be considered when developing pro-angiogenic therapeutic strategies.

### 5.4 The Inflammatory Context

The inflammatory environment substantially influences defective angiogenesis in CTEPH. In animal models examined by Alias et al. [77], the expression of plasminogen activator inhibitor-1 (*PAI-1*), a known angiogenesis suppressor, was elevated in the obstructive pulmonary vasculature. A sustained inflammatory response can restrict angiogenesis by elevating inhibitor levels and impairing normal cellular functions critical for vascular repair. Moreover, Neto-Neves et al. [78] reported that the endothelial proliferative response to vascular endothelial growth factor (*VEGF*) was impaired in their rat model, leading to phenotypic changes resembling those of CTEPH. The coupling of impaired angiogenesis with inflammation in these models effectively resembled the clinical scenario observed in human CTEPH, suggesting a critical nexus that influences disease pathology.

## 6. Conclusion

**Fig. 3.**
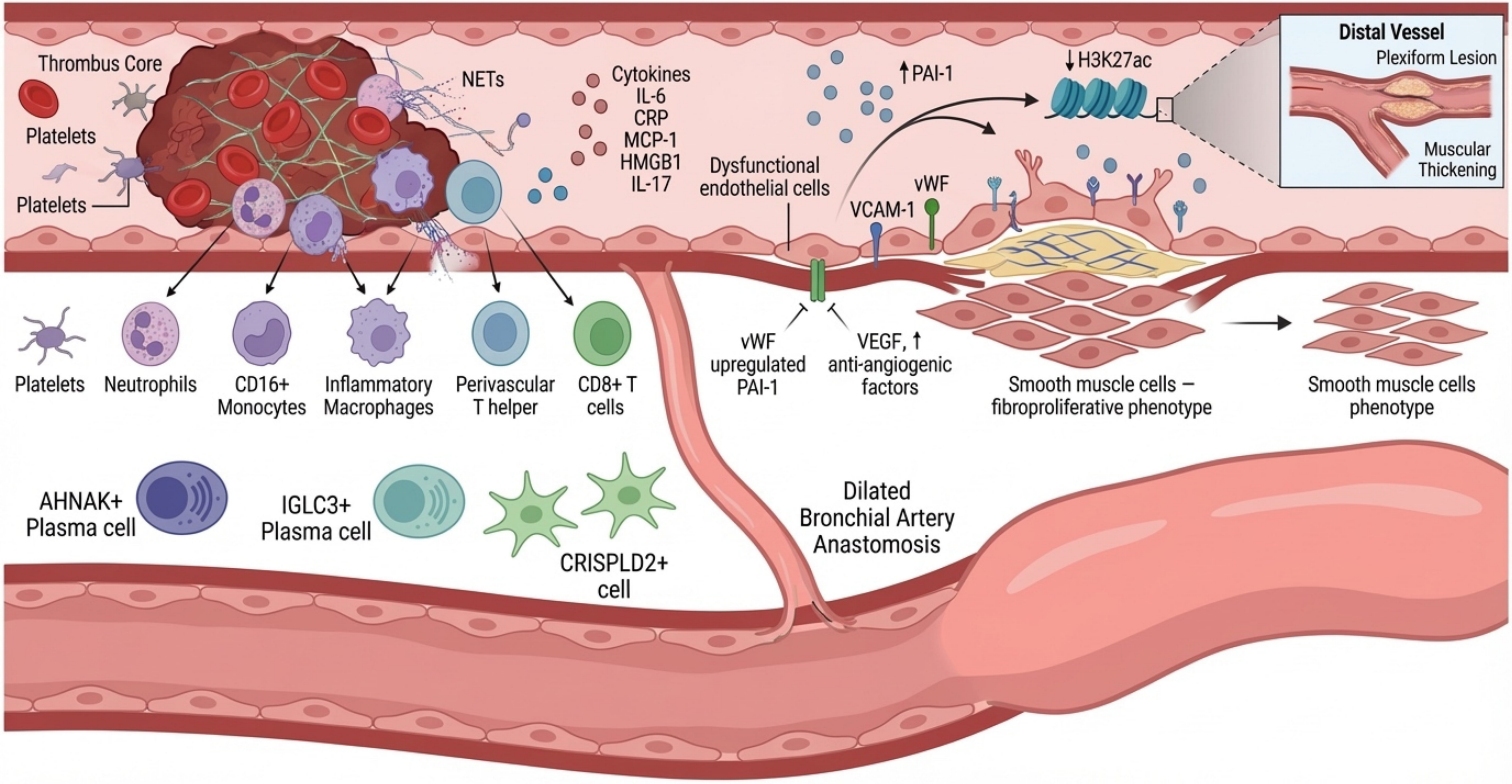
**Cellular and molecular landscape of the CTEPH pulmonary vasculature**.

Chronic thromboembolic pulmonary hypertension cannot be sufficiently explained by passive mechanical obstruction alone. Increasing evidence has demonstrated that CTEPH is a dynamic, biologically active disease driven by sustained interactions among inflammation, endothelial dysfunction, and impaired angiogenesis. After an acute thromboembolic event, persistent inflammatory activation and maladaptive immune responses disrupt physiological thrombus resolution, leading to fibrotic transformation of embolic material and progressive pulmonary vascular remodeling (Fig. 3). Endothelial cells are central orchestrators of disease progression, responding to altered shear stress and inflammatory stimuli with a pro-thrombotic, pro-inflammatory phenotype marked by *NF-κB*–dependent signaling, epigenetic reprogramming, and dysregulated von Willebrand factor expression. Concurrently, defective angiogenesis, resulting from an imbalance between pro- and anti-angiogenic mediators within an inflammatory microenvironment, impedes effective vascular recanalization and perpetuates pulmonary hypertension, even after proximal obstruction is removed.

Recognizing CTEPH as an inflammation–angiogenesis disorder has significant clinical implications. This perspective offers a mechanistic explanation for disease progression, distal vasculopathy, and postoperative residual pulmonary hypertension while also identifying novel therapeutic avenues beyond anticoagulation and surgical intervention. Approaches that target immune-vascular interactions, including agents designed to dissolve *NETs* (e.g., *DNase I*), *PAD4 *inhibitors, or therapies targeting leukocyte-derived extracellular vesicles (LEVs) that promote microthrombosis, as well as strategies targeting broader immune–vascular interactions, restore endothelial homeostasis, and modulate angiogenic pathways may serve as promising adjunctive strategies to improve long-term outcomes in patients with CTEPH. Emerging preclinical evidence supports several specific therapeutic avenues. Riociguat, a soluble guanylate cyclase stimulator, has been shown to enhance circulating endothelial progenitor cell numbers and angiogenic gene expression, promoting beneficial neovascularization [76]. Given the emerging role of extracellular vesicles in maintaining the inflammatory microenvironment, therapies targeting leukocyte-derived EV release or uptake represent a promising frontier. PAD4 inhibitors, which target peptidylarginine deiminase 4 involved in *NET* formation, have been correlated with CTEPH severity and right heart dysfunction, and *DNase I *administration has demonstrated efficacy in alleviating pulmonary arterial hypertension in experimental models by degrading *NET* scaffolds. Future research integrating high-resolution single-cell analyses, epigenetic profiling, and translational models is essential to refine risk stratification, identify biomarkers of disease activity, and develop mechanism-based therapies. An advanced understanding of the inflammatory and angiogenic drivers of CTEPH may shift the current paradigm from purely mechanical correction toward comprehensive disease modification.
